# Computational Study of Graphene–Polypyrrole Composite Electrical Conductivity

**DOI:** 10.3390/nano11040827

**Published:** 2021-03-24

**Authors:** Oladipo Folorunso, Yskandar Hamam, Rotimi Sadiku, Suprakas Sinha Ray

**Affiliations:** 1 Department of Electrical Engineering, French South African Institute of Technology (F’SATI), Tshwane University of Technology, Pretoria 0001, South Africa; HamamA@tut.ac.za; 2 Centre for Nanostructures and Advanced Materials, DSI-CSIR Nanotechnology Innovation Centre, Council for Scientific and Industrial Research, Pretoria 0001, South Africa; 3 École Supérieure d’Ingénieurs en Électrotechnique et Électronique, Cité Descartes, 2 Boulevard Blaise Pascal, Noisy-le-Grand, 93160 Paris, France; 4 Department of Chemical, Institute of NanoEngineering Research (INER), Metallurgy and Material Engineering, Tshwane University of Technology, Pretoria 0001, South Africa; sadikur@tut.ac.za; 5 Department of Chemical Sciences, University of Johannesburg, Doornfontein, Johannesburg 2028, South Africa

**Keywords:** graphene, Monte Carlo, electrical conductivity, polypyrrole, numerical model

## Abstract

In this study, the electrical properties of graphene–polypyrrole (graphene-PPy) nanocomposites were thoroughly investigated. A numerical model, based on the Simmons and McCullough equations, in conjunction with the Monte Carlo simulation approach, was developed and used to analyze the effects of the thickness of the PPy, aspect ratio diameter of graphene nanorods, and graphene intrinsic conductivity on the transport of electrons in graphene–PPy–graphene regions. The tunneling resistance is a critical factor determining the transport of electrons in composite devices. The junction capacitance of the composite was predicted. A composite with a large insulation thickness led to a poor electrochemical electrode. The dependence of the electrical conductivity of the composite on the volume fraction of the filler was studied. The results of the developed model are consistent with the percolation theory and measurement results reported in literature. The formulations presented in this study can be used for optimization, prediction, and design of polymer composite electrical properties.

## 1. Introduction

Uniform dispersion of nanofillers in a polymer matrix improves the electrical conductivity, thermal conductivity, mechanical properties, and chemical stability of the composite [1,2]. Carbon black, carbon nanotubes, and graphene have been used to control the electrical conductivities of conducting and nonconducting polymers [1,3,4]. The flexible control of the properties of nanocomposites enables their diverse applications in numerous sectors, including energy, control engineering, health, aviation, textiles, and electronics [5,6,7,8].

Composites with graphene are more efficient than other carbon material composites owing to its astounding electrical conductivity, flexibility, simple synthesis methods, and super-electrochemical action when it is composited with conducting polymers [9,10,11]. The unique morphology, surface area, and electrical conductivity provide various applications of graphene in various fields, particularly in energy storage. During the electrochemical action of graphene, the combination of its excellent mechanical strength and high aspect ratio are expected to aid the resulting composite porosity, thereby limiting the cracking and fracture of the electrode [12]. In addition, the chemical and thermal stabilities of graphene are advantageous in the protection from damage of the highly porous electrode [13]. In electrolyte diffusion, graphene, as a good electrical conductor, is an excellent current collector for the passage of ions within the pores of the electrochemical electrodes [14]. PPy is a good conducting polymer, promising, as a super-electrode, for the manufacturing of supercapacitors and batteries. PPy, as a conducting polymer, can be simply synthesized, has a low cost and good mechanical and thermal stabilities, and is environmental benign [15,16]. The composite of graphene–PPy is envisaged to be the next-generation energy harvester [17]. Due to various parameters on which graphene-PPy or other polymer-composites depend, their laboratory productions cannot exhibit the explicit properties of the composites, and thus the modeling approach is a vital tool for their proper analysis [18].

**Figure 1:**
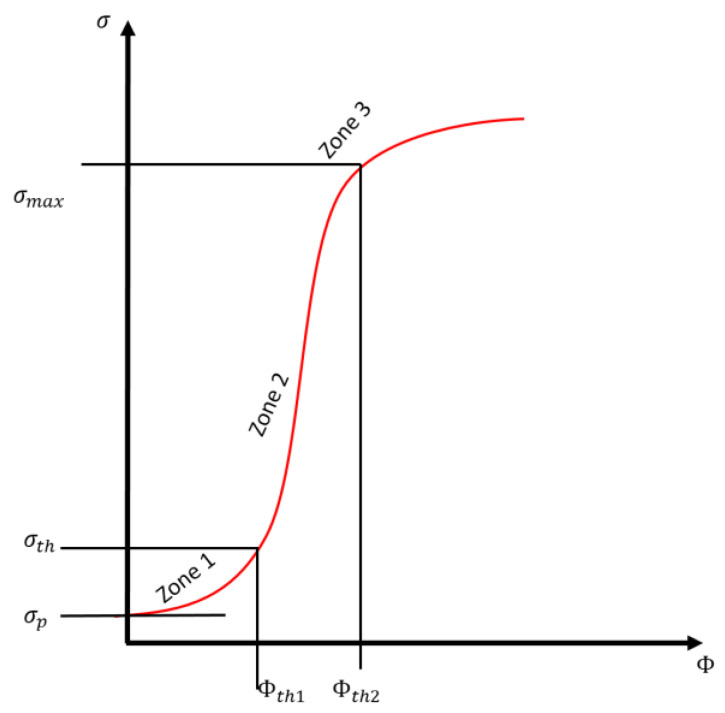
*S*-like percolation curve of the polymer composite electrical conductivity, where *σ_p_* is the conductivity of the polymer, *σ_th_* is the conductivity at the percolation threshold, and *ϕ_th1_* and *ϕ_th2_* are the lower and upper volume fractions, respectively.

By considering the experimental behaviors of polymer composites, the transition of the composite from insulator to semiconductor/conductor can be mathematically described by the percolation theory with respect to the volume fraction of the filler [19,20,21]. The critical parameter determining the conductivity is the volume fraction of the fillers, Φ. Figure 1 presents the *s*-like shape of the percolation curve for polymer composites. As shown in Figure 1, zone 1 depicts the percolation threshold of the system, i.e., a state in which the matrix system starts to conduct or where its initial conduction begins to noticeably change. In zone 2, the conduction changes linearly with the volume fraction. In zone 3, the conductivity is constant with the increase in the volume fraction. However, the calculation of the electrical conductivities of polymer composites is challenging because of several factors, such as filler size, shape, orientation, aspect ratio, and matrix potential barrier, which determine the overall properties of the composite [22,23]. The threshold occurs at given volume fraction. The electrical conductivity of the material increases sharply to a point where it becomes independent on the volume fraction [22].

To mitigate the challenges in quantifying the various factors determining the electrical conductivity and other properties of graphene–PPy, the Monte Carlo simulation approach (MCSA) is employed in this study. Fang et al. [23] predicted the percolation threshold and calculated the electrical conductivities of polymer–carbon-nanotube composites (PCNTCs) using the MCSA. The ambiguities in the simulation approach were reduced by the use of the MCSA. Yu et al. [24] studied the effects of the tunneling resistance on the electrical conductivity of the PCNTC using the MCSA. The computational efficiency of Yu et al. [24] was attributed to the Monte Carlo simulation method. The total electrical conductivity of polymer–carbon nanotube was obtained by Gong et al. [25]. The carbon nanotube percolation was in good agreement with the measurement results. Li et al. [26] carried out a study on the electrical conductivity of PCNTC, where the contact resistance between the matrix and filler was analyzed by the MCSA. The MCSA is an effective approach for the calculation of the electrical conductivities of polymer composites [20,24].

Moreover, to predict the electrical properties of polymer composites, their tunneling and intrinsic resistances must be evaluated. The resistivity of single-walled carbon nanotubes (CNTs) varies between $5.1\times 10^{-8}\;\mathrm{and}\;5.8\times 10^{-2}\;\Omega m$ [27], while the electrical contact resistance of CNTs has a threshold of $10^{6}\;\Omega$ [28]. The total contact resistance between nickel and monolayer graphene was ~790 ±300 Ω [29]. The tunneling resistance or contact resistance is crucial for the electrical performances of polymer composites, because it determines the electrical transport in the composite. Moreover, proper understanding of the effect of the tunneling resistance in polymer composites could enable further electrochemical, sensor, and electronic applications. If the polymer thickness between the layers of the fillers is too large, the composite would exhibit a low electrical conductivity. More so, the Fermi level of graphene can be subjected to change via an external electric field, due to its atomically thin nature and approximately zero density of state. That is, the interfacial properties of graphene are susceptible to variation, depending on the thickness of the insulating film and the bias voltage [30]. In addition, the electrical conductivity of composite with respect to interfacial junction, can be controlled by thermionic-charge-injection process. Extensive studies on the impact of thermionic-charge-injection on the contact resistance of 2-dimensional (2D) and 3-dimensional (3D) materials have been reported in literature [31,32,33].

Therefore, for graphene nanocomposites, maximum contact resistance, the effect of polymer thickness on the capacitance behavior of the composite, and the contribution of the intrinsic resistance of fillers to the overall electrical conductivities of the resulting composites, are important parameters which must be evaluated. These aforementioned parameters and others are investigated in this study. In addition, a prediction based on the simulations of the contact resistance of PPy on graphene is presented. The developed model can be used to quantify the electrochemical behavior of the polymer composite.

In this study, a Monte-Carlo is used in the computation of electrical conductivity model developed to investigate the associated percolation in graphene–PPy. The model incorporated a reported contact model [34] and McCullough equation [35] to estimate the electrical conductivity of the graphene–PPy composite. The input parameters are the thin-film insulator dielectric constant, bias voltage, tunnel potential barrier, thickness of the PPy, permittivity of free space, graphene cross-sectional area, electronic charge, and PPy and graphene intrinsic electrical conductivities. All other factors that determine the electrical conductivities of the polymer composites were included in the MCSA code. The results of the model were compared to measurement results [36,37]. The model data were consistent with the experimental values. The electrical properties of the graphene–PPy composites were precisely and efficiently predicted. The model could be also used for the investigation of electrical properties of other polymer composites/nanocomposites.

## 2. Electrical Conductivity of Graphene-PPy Composite

The analysis of the variation in the conductivity level of a polymer with included two-dimensional materials is complex. A simulation approach that can produce a random distribution of fillers in polymers is essential for the modeling of the electrical conductivities of polymer composites. A statistical computation based on the generation of random parameters to obtain numerical results is referred to as the Monte Carlo model. The computational steps employed in this study involve the generation of a random graphene network distribution using the MCSA, modeling of the sheet-to-sheet resistance of the graphene in the composite, inclusion of the filler intrinsic resistance, and application of the rules of mixtures to quantify the electrical conductivity of the composite.

**Figure 2:**
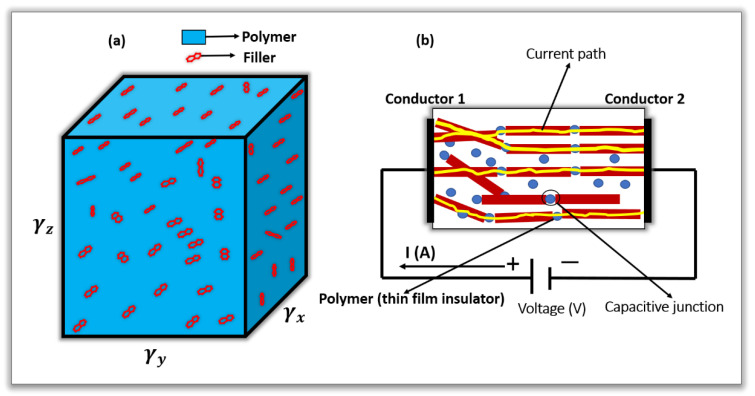
(**a**) RVE for the graphene–PPy nanocomposite and (**b**) resistive network of the randomly dispersed graphene in polypyrrole. The current path is formed when the bias voltage is larger than the tunneling barrier potential.

A three-dimensional percolation network was created by a Monte Carlo model to predict the threshold of the composite. We developed a cubic representative volume element (RVE) of the graphene-PPy composite and obtained a conductivity equation. It was assumed that the electrical resistance of the graphene–PPy composite can be modeled by the resistance of the graphene percolation networks and electrical conductivity contribution of the polymer in the RVE. As shown in Figure 2, the cubic RVE of the graphene-PPy has a side length of γ and is randomly filled with distributed graphene sheets. The graphene is represented by rectangular conducting bars with diameters of D and lengths of $\gamma_{g}$ [21,38].

The dimensions of the cuboid RVE are $\gamma_{x}\;\times \;\gamma_{y}\;\times \;\gamma_{z}$. $\delta_{i}\;\;\mathrm{and}\;\theta_{i}$ are the polar and azimuthal angles, representing the filler orientations. If the start and end points of the cuboid are $\gamma_{xi},\;\gamma_{yi},\;\;\mathrm{and}\;\gamma_{zi}$ and $\gamma_{xj},\gamma_{yj},\;\;\mathrm{and}\;\gamma_{zj}$, respectively, the cluster representative of the network is [39,40]:

(1) $$\left(\begin{array}{c} \gamma_{xi}\\ \gamma_{yi}\\ \gamma_{zi} \end{array}\right)=\;\left(\begin{array}{c} \gamma \;\times rnd\\ \gamma \;\times rnd\\ \gamma \;\times rnd \end{array}\right)$$

(2) $$\left(\begin{array}{c} \begin{array}{c} \gamma_{xj}\\ \gamma_{yj} \end{array}\\ \gamma_{zj} \end{array}\right)=\;\left(\begin{array}{c} \gamma_{xi}\\ \begin{array}{c} \gamma_{yi}\\ \gamma_{zi} \end{array} \end{array}\right)+\;\gamma_{g}\left(\begin{array}{c} \sin\left(\delta_{i}\right)\\ \begin{array}{c} \sin\left(\delta_{i}\right)\\ \cos\left(\delta_{i}\right) \end{array} \end{array}\right)\left(\begin{array}{c} \cos\;\left(\theta_{i\;}\times rnd\right)\\ \begin{array}{c} \sin\;\left(\theta_{i\;}\times rnd\right)\\ \sin\left(\frac{\pi }{2}\;\times rnd\right) \end{array} \end{array}\right)$$

(3) $$\left(\begin{array}{c} \theta_{i}\\ \delta_{i} \end{array}\right)=\;\left(\begin{array}{c} 2\pi \\ \cos^{-1}\left(2\;\times rnd-1\right) \end{array}\right)$$

(4) $$\xi \left(\gamma_{g};a,\;b,c\right)=\;\{\begin{array}{cc} \frac{b}{a}{\left(\frac{\gamma_{g}-c}{a}\right)}^{b\;-1}\exp\left(-{\left(\frac{\gamma_{g}-c}{a}\right)}^{b}\right) & \gamma_{g}\;>c\\ 0 & \gamma_{g}<\;c \end{array}$$

where rnd is a random value (0,1), $\gamma_{g}$ is the length of the nanorod, and a, b, and c are the Weibull scaling, shaping, ξ is the probability distribution function, and locator parameters, respectively. The characterizations of the filler length and its diameter were performed using the Weibull distribution (Equation (4)) [41]. The filler is periodically arranged in the directions $\gamma_{x}$ and $\gamma_{y}$.

**Figure 3:**
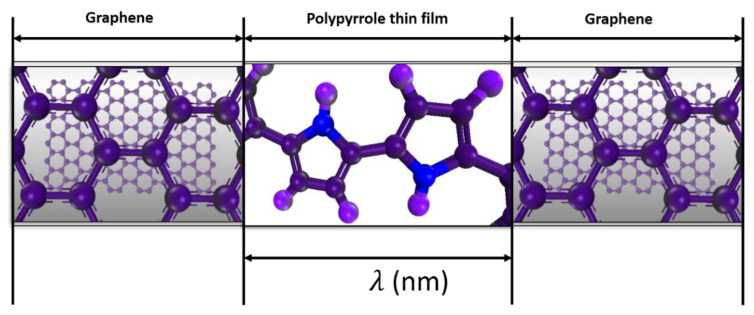
Graphene–PPy composite electrode.

Two types of resistance determine the electrical conductivities of nanocomposites [39,40], i.e., the tunneling and intrinsic filler resistances. If the filler is evenly dispersed in the matrix at a low concentration, the transport of electrons in the composite is determined by the tunneling/contact resistance. The tunneling resistance is formed when separating distances are created between the sheets of the filler by the polymer [26,34]. Accordingly, for an effective and reliable predictive model, the tunneling and intrinsic resistance of the filler in the composite are considered. Figure 3 shows a schematic of the graphene–PPy electrode, separated by a thin film of PPy.

According to Yu et al. [24], the tunneling resistance of a polymer composite is a function of the thickness, dielectric material of the polymer insulating layer, and orientation of the fillers. As shown in Figure 3, the polymer separation distance is λ, while the effective area of the formed contact is $A_{g}$. The tunneling resistance is created when the separating distance is smaller than the diameter of the graphene. The tunneling resistance of the composite is estimated using the Simmons’s equation [34]. The current density, J, is [24,26]:

(5) $$J=\;J_{0}\left[\left(\varphi_{1}\exp^{-\tau \sqrt{\varphi_{1}}}\right)-\left(\varphi_{1}+V\right)\exp^{-\tau \sqrt{\varphi_{1}+\;V}}\;\right],$$

where J is the current density, $\varphi_{1}$ is the mean tunnel potential barrier, V is the bias potential across the sheets of graphene, and $J_{0}$ and μ are values, which were determined by

(6) $$J_{0}=\;\frac{q^{2}}{2\pi h{\left(\Delta \lambda \right)}^{2}};\tau =\frac{4\pi \sqrt{2m_{e}q}\Delta \lambda }{h},$$

where $\Delta \lambda =\lambda_{2}-\lambda_{1}$, h is Planck constant, $m_{e}$ is mass of electron, and q is electronic charge. The mean potential barrier is calculated by

(7) $$\varphi_{1}=\;\varphi_{0}-V\left(\lambda_{1}+\lambda_{2}\right){\left(2\lambda \right)}^{-1}-\;\left(5.75{\left(\beta \Delta \lambda \right)}^{-1}\right)\ln\left(\frac{\lambda_{2}\left(\lambda -\lambda_{1}\right)}{\lambda_{1}\left(\lambda -\lambda_{2}\right)}\right)$$

where λ is the thickness of the PPy film and $\varphi_{0}$ is the height of rectangular barrier voltage. It was assumed that the barrier voltage is larger than the voltage across the insulating polymer film. Therefore, the barrier limits [34], $\lambda_{1}$ and $\lambda_{2}$, are:

(8) $$\lambda_{1}=\frac{6}{\beta \varphi_{o}};\lambda_{2}=\lambda \left[1-\;\frac{46}{\left(3\varphi_{o}-2V\right)\beta \lambda +20}\right]+\;\lambda_{1}.$$

The electronic charge, q, is the product of the capacitance, C, developed at the junction and applied bias voltage. The voltage across the insulating film is [26]:

(9) $$V=\;\frac{q}{C}=\;\frac{q\lambda }{\beta A_{g}\varepsilon_{0}},$$

where β is the insulator dielectric constant, $A_{g}$ is the graphene cross-sectional area, $\varepsilon_{0}$ is the permittivity of free space, and $\varphi_{0}$ is the height of rectangular barrier potential. The resistance between the sheets of the filler is estimated by:

(10) $$R_{s-s}=\frac{V}{JA_{g}}=\frac{q\lambda }{\beta A_{g}^{2}\varepsilon_{0}\left(\;J_{0}\left[\left(\varphi_{1}exp^{-\mu \sqrt{\varphi_{1}}}\right)-\left(\varphi_{1}+V\right)\exp^{-\mu \sqrt{\varphi_{1}+\;V}}\;\right]\right)}.$$

According to Equation (10), the tunneling resistance is estimated to be $R_{s-s}$.

The intrinsic resistance of the filler was modeled by considering the law of resistance:

(11) $$R_{g}={\left(\frac{\sigma_{g}\pi D^{2}}{4\gamma_{g}}\right)}^{-1},$$

where $R_{g}$ is the intrinsic graphene resistance, D is the diameter of the graphene, and $\gamma_{g}$ is the graphene length.

The effective electrical conductivity of the graphene dispersed in the PPy is obtained by the McCullough equation [35], based on the rule of mixtures and transport properties of homogenous mixtures:

(12) $$\sigma_{c}={\;\Phi }_{f}\sigma_{T}+{\;\Phi }_{PPy}\sigma_{PPy}-\;\frac{\zeta_{f}\Phi_{PPy}\Phi_{f}{\left(\sigma_{T}\;-\;\sigma_{PPy}\right)}^{2}}{\Phi_{PPy,i}\sigma_{PPy}+{\;\Phi }_{f,i}\sigma_{T}},$$

where $\sigma_{c}$ is the composite electrical conductivity, $\Phi_{PPy}$ is the PPy volume fraction, $\Phi_{f}$ is the filler volume fraction, $\sigma_{PPy}$ is the PPy electrical conductivity, and $\zeta_{f}$ is the filler length factor $\left(0\leq \zeta_{f}\leq 1\right)$. The conducting network electrical conductivity is:

(13) $$\sigma_{T}=\;\frac{4\gamma_{g}}{\pi D^{2}R_{T}};\;R_{T}=R_{s-s}+R_{g},$$

while

(14) $$\Phi_{f,i}=\;\left(1-\zeta_{f}\right)\Phi_{f}+\zeta_{f}\Phi_{PPy}$$

and

(15) $$\Phi_{PPy,i}=\;\left(1-\zeta_{f}\right)\Phi_{PPy}+\zeta_{f}\Phi_{f}.$$

The summary of the parameters used in the simulation are given in Table 1.

## 3. Results and Discussion

In this section, we discuss the (i) effect of the polymer insulation thin film on the tunneling resistance; (ii) relationship between the intrinsic resistance and tunneling resistance; (iii) composite capacitance; (iv) validation of the model with experimental measurements; (v) tunneling resistance and electrical conductivity; (vi) effect of the aspect ratio on the composite electrical conductivity; and effect of the graphene electrical conductivity on the composite.

**Figure 4:**
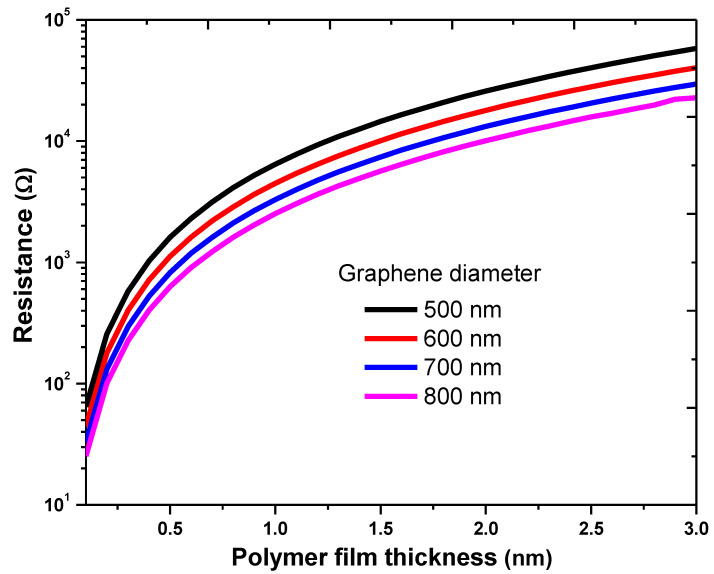
Tunneling resistance as a function of the diameter and insulation thickness.

For the determination of tunneling resistance, the graphene height of rectangular potential barrier is set to 4.6 eV [42], the PPy dielectric constant is $10^{5}$ [43], and the permittivity of free space is $8.854\times 10^{-12}\;C/\mathrm{Vm}$ [44]. Figure 4 shows the calculated results for the tunneling resistance of graphene–PPy–graphene. The thickness of the PPy was 0.1–3 nm, while the diameter of the graphene was in the range of 500–800 nm [45]. Figure 4 shows that the junction resistance is a function of the polymer thickness and diameter of the filler. At a polymer thickness of 1 nm, the tunneling resistances were 6.5, 4.5, 3.3, and 2.52 kΩ at graphene diameters of 500, 600, 700, and 800 nm, respectively. The resistance increases with increasing thickness. However, the resistance decreased with the increase in the graphene diameter. The variation in the dielectric constant of the polymer led to a noticeable increment in the tunneling resistance. These results agree with the calculations of Li et al. [26], Yu et al. [24], and Simmons [34]. In this regard, the insulating thickness of the polymer must be very small for electrons to penetrate the low-conductivity junctions. Thus, a smaller insulating thickness implies a higher electron tunneling in the polymer composite, and hence a higher electrical conductivity.

**Figure 5:**
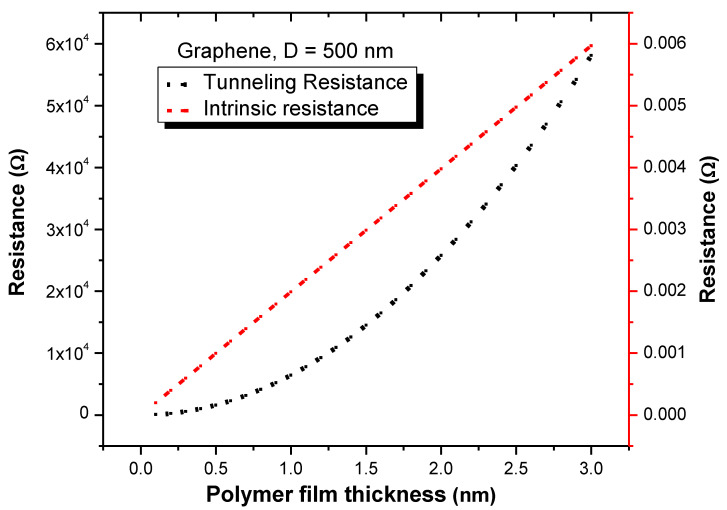
Intrinsic and tunneling resistances as a function of insulation thickness.

Figure 5 compares the intrinsic and tunneling resistances for a total insulation thickness of 3 nm and graphene diameter of 500 nm. When the insulation thickness was extremely small, the intrinsic resistance of the graphene was considerably negligible. However, at an insulation thickness of 0.4 nm, the tunneling and intrinsic resistances were approximately 1 kΩ and $7.9\;\times 10^{-4}$ Ω. At an insulation thickness of 2 nm, the intrinsic and tunneling resistances were 258.8 kΩ and $3.98\;\times 10^{-3}$ Ω, respectively. Thus, the intrinsic resistance of the graphene, though negligible, has a small proportional effect to the insulation thickness of the polymer thin film. Furthermore, the tunneling resistance increased with the insulation thickness. This result agrees with the results of Gong et al. [46] and Yu et al. [24].

**Figure 6:**
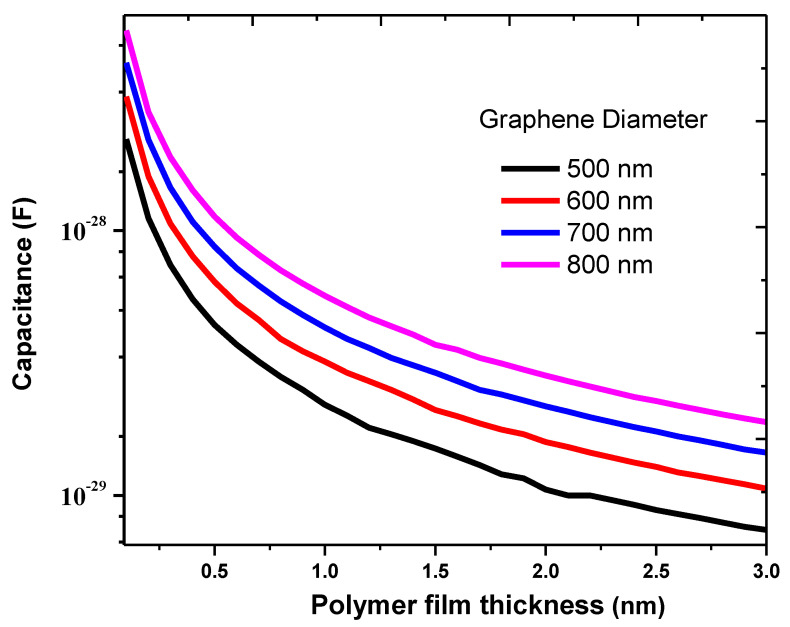
Capacitance of the junction as a function of graphene diameter and insulation thickness.

In addition, the specific capacitances of the graphene–PPy composites with different insulation thicknesses of 0.1–3 nm and graphene diameters of 500–800 nm are presented in Figure 6. The insulation thickness affects the capacitance behavior and electrical conductivity of the composite. The specific capacitance of the graphene–PPy increased with decrease in insulation thickness. This implies that, at a very small insulation thickness, the graphene–PPy composite electrode has a high capacity for ionic transportation during electrochemical action. A very small insulation thickness would increase the electron mobility because of the reduced resistance at the graphene–PPy–graphene junctions. The experimental results of Zhang et al. [47] and Chang et al. [48] agree with the results of this study.

**Figure 7:**
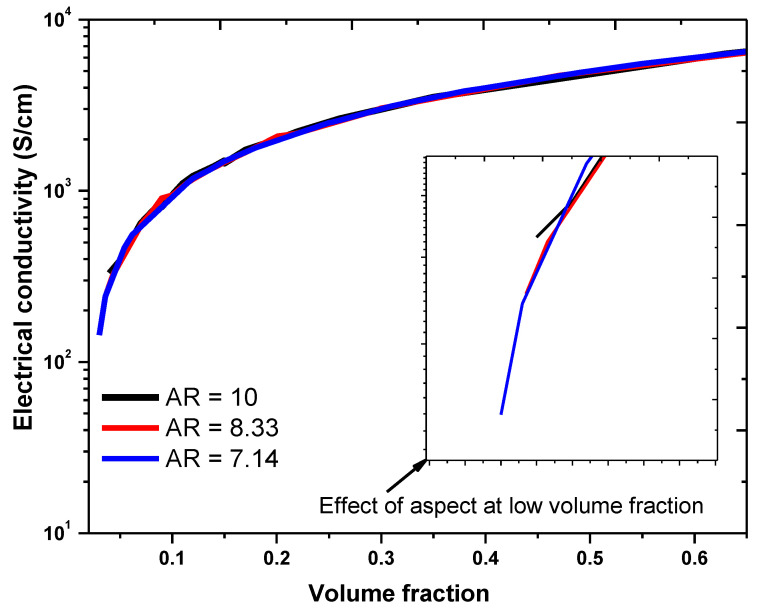
Electrical conductivity of the graphene–PPy composite at different aspect ratios.

The influence of the aspect ratio $\left(\frac{\gamma_{g}}{D}\;\right)$ of the graphene used in this study on the electrical conductivity of the graphene–PPy composite is shown in Figure 7. For an insulation thickness of 0.9 nm, graphene length of 5 µm, matrix and graphene conductivity of 1 S/cm and 10^3^ S/m, the aspect ratio was varied by employing a graphene diameter of 500–700 nm. The results indicate that the aspect ratio had a considerable influence on the formation of the conducting path of the composite. As shown in Figure 7, the composite with the smallest diameter requires a high-volume concentration of graphene to form the desired conducting path. Moreover, the conducting path increased with the graphene diameter. In addition, the aspect ratio had a significant influence on the effective conductivity of the composite at low volume fractions. However, with the increase in volume fraction, the composite conductivities at different aspect ratios become saturated and proportional to the aspect ratio. The composite electrical conductivity was less sensitive to the aspect ratio beyond the percolation region. These results are consistent with those of other studies [39,40,49].

**Figure 8:**
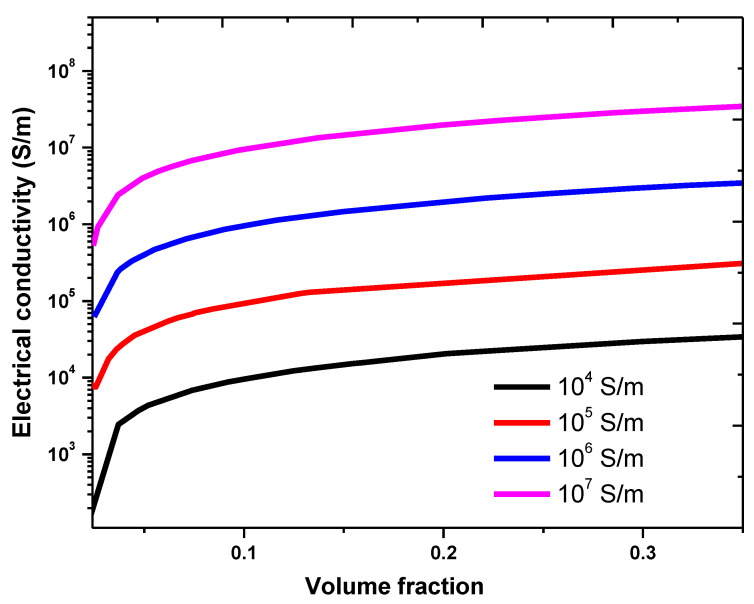
Electrical conductivity of the graphene–PPy composite for different graphene volume fractions and dc electrical conductivities.

The effect of the graphene intrinsic conductivity was also investigated, as shown in Figure 8. The selected parameters were graphene length = 2 µm, graphene diameter = 500 nm, matrix conductivity = 100 S/m, and insulation thickness = 0.9 nm. The effect of the intrinsic electrical conductivity of graphene was studied in the range of $10^{4}-10^{7}\;S/m$ [50]. The composite electrical conductivity significantly increased with the graphene electrical conductivity. The increase in the effective conductivity of the composite with the increase in the filler conductivity is crucial for device applications, such as supercapacitors, sensors, batteries, and solar cells [40,51]. The calculation shows that the percolation threshold is independent on the filler conductivity. This result agrees with the literature results [40,46,52].

**Figure 9:**
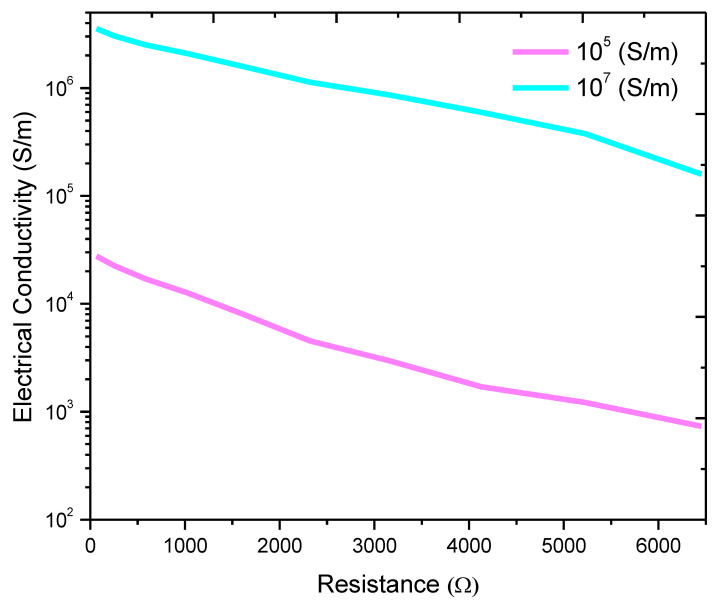
Tunneling effect on the electrical conductivity of the graphene–PPy composite.

The contribution of the tunneling resistance to the electrical conductivity of the graphene–PPy composite was analyzed by considering the insulation thickness in the range of 0.1–1 nm, intrinsic PPy conductivity of 10 S/m, volume fraction of approximately 0.04, graphene diameter of 500 nm, and graphene conductivities of $10^{5}$ and $10^{7}$ S/m. As shown in Figure 9, the effective electrical conductivity of the composite is inversely proportional to the insulation thickness. In other words, for electron tunneling in the composite, the insulation thickness of the PPy must be small. The composite experiences a low barrier to electron transport when the insulating thickness is relatively small. This result agrees with the numerical simulation by Payancdehpeyman et al. [53]. However, graphene with a very high intrinsic electrical conductivity can create a negligible tunneling effect owing to its ability to rapidly form a conducting network.

**Figure 10:**
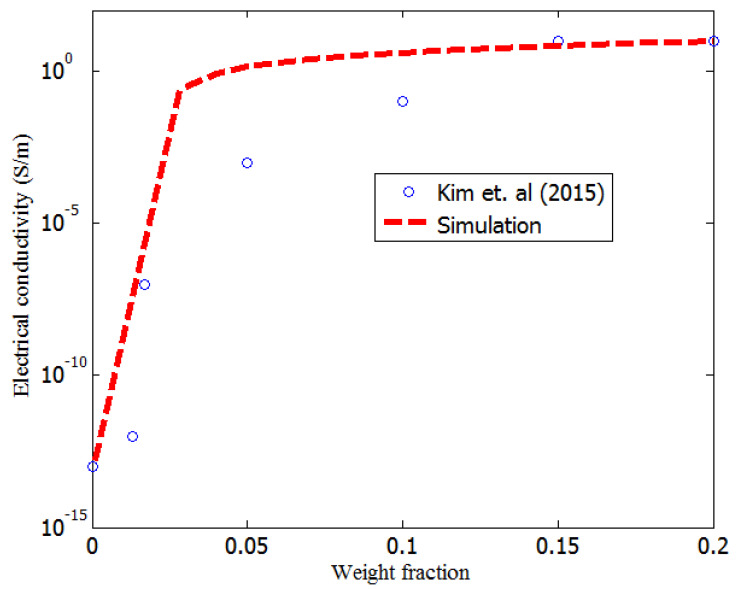
Comparison of the simulation results to measurement results (Kim et al.) [37].

Furthermore, in this study, the developed model was validated by reported measurements. Kim et al. [37] carried out a homogenous dispersion of multiple fillers (graphene, carbon black, and CNTs) on cyclic butylene terephthalate (CBT), obtained by powder mixing and in-situ polymerization (8 data points). The synthesis methods were appropriate for homogenous dispersion of fillers in the polymer matrix. The electrical conductivities of the CBT and graphene nanoplatelets were $8.50\;\times 10^{-14}\;S/m$ and $10^{5}\;S/m$ [37], respectively. The diameter, insulation thickness, and length of the carbon black [37] were 5 μm, 3 nm, and 6 nm, respectively. Figure 10 compares the results of the developed model in this study to the measurement results of Kim et al. [37]; they agree reasonably well.

**Figure 11:**
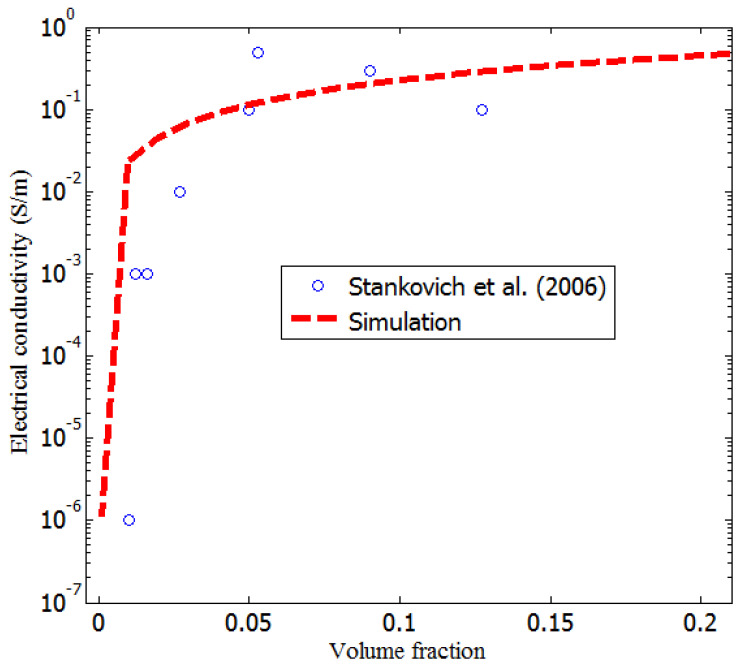
Comparison of the simulation results to measurement results (Stankovich et al.) [36].

To further confirm the robustness of the model, the results of a set of experimental electrical conductivity measurements of the graphene-polystyrene (graphene–PS) composite (10 data points), carried out by Stankovich et al. [36], were compared to the results of the developed model. The solvent mixing method was employed by Stankovich et al. [36] to uniformly disperse the graphene in the polymer matrix. After the filtration of the mixture of the composite from the solvent, it was washed with 200 mL of methanol. At a filler volume fraction of 0.1%, the composite electrical conducting path was formed. For the developed model, the diameter, length, insulation thickness, and initial conductivities of graphene and PS were $50\;\mu m,\;2\;\mathrm{nm},\;3\;\mathrm{nm},\;10^{2}\;S/m,$ and $10^{-15}\;S/m$, respectively. Figure 11 compares the electrical conductivity measurement results reported by Stankovich et al. [36] to those of the model developed in this study. The obtained results agree reasonably well with the measurement results [36].

In summary of this section, an excellent electrically conductive material such as graphene has been shown to be suitable for the enhancement of the electrical properties of polymer (polypyrrole). However, the performances of this composite are largely dependent on the thickness of the polymer. The tunneling of electrons between the sheets of the conductor is a function of the thickness of the polymer film. From the modeling results, the effects of filler diameter and matrix thickness has been shown to have varying impact in the tunneling resistance of the composite. For example, a low equivalent resistance is required to obtain a high conductive and high capacitive supercapacitor [54]. Therefore, a supercapacitor-based polymer-composite must be manufactured with filler having large diameter, and small matrix thickness. To experimentally control the insulating thickness in a polymer-composite, the synthesis method must be appropriately chosen and adopted [55,56]. More so, the insulating thickness has an inverse effect on the capacitance and the electrical conductivity nanocomposite. It was further noted that the intrinsic resistance of the filler has almost a negligible effect on the overall conductivity of the nanocomposites. Furthermore, the model experiences some limitation due to the length chain factor ($\zeta_{f}$) introduced by McCullough. The growth of the conductive network can be dampened or overestimated depending on the value of the $\zeta_{f}$. The accuracy of Simmons equation for direct tunneling is reported to depend on the thickness of the insulating film (>1 nm) and the barrier height (3 eV) [57]; these conditions were fulfilled in this study.

## 4. Conclusions

This study considered the use of the Simmons and McCullough equations, in conjunction with the MCSA, to calculate the contact resistance in the graphene–PPy composite and electrical conductivities of its constituents. The results of the developed model agreed well with the results of previous measurements. The barrier junction of the polymer composite strongly depended on the filler diameter and polymer thickness. With the increase in insulation thickness, the intrinsic conductivity of the filler had a negligible effect on the effective conductivity of the composite. In addition, the capacitive behavior of the composite showed that the graphene–PPy is a good electrochemical composite, owing to the large diameter of the graphene and very small thickness of the PPy. The influences of aspect ratio, graphene intrinsic conductivity, and volume fraction on the electrical conductivity of the graphene–PPy composite were investigated. The consistency of the model results with those of measurements and previous studies shows its suitability for the theoretical evaluation of polymer composite electrical properties. Further experimental and theoretical studies on the feasibility of using graphene–PPy composites for solar cell and battery applications are required.

## Figures and Tables

**Table 1:** The model parameters.

Parameters	Meaning	Unit
J	Current density	$A/{\mathrm{cm}}^{2}$
$\lambda_{1},\lambda_{2}$	Limits of insulating barriers at Fermi level	nm
λ	Thickness of polymer insulating film	nm
τ	Decay parameter	1/ÅV
$\varphi_{1}$	Mean barrier height	eV
$\varphi_{0}$	Height of rectangular barrier	eV
V	Bias voltage	V
β	Insulator dielectric constant	
h	Planck constant	Js
$m_{e}$	Mass of electron	kg
q	Electronic charge	C
$\varepsilon_{0}$	Permittivity of free space	F/m
C	Junction capacitance	F
$A_{g}$	Filler cross-sectional area	${\mathrm{nm}}^{2}$
$\Phi_{f}$	Filler volume fraction	
$\Phi_{PPy}$	Polymer volume fraction	
$\sigma_{PPy}$	Polymer conductivity	S/m
$\sigma_{g}$	Filler conductivity	S/m
$\zeta_{f}$	Filler length factor	nm
$\gamma_{g}$	Filler length	nm
$\gamma_{x},\;\gamma_{y},\;\gamma_{z}$	Dimension of cuboid RVE	${\mathrm{nm}}^{3}$
$\delta_{i}$ and $\theta_{i}$	Polar and azimuthal angles	
rnd	Random values	
D	Filler diameter	nm
ξ	Weibull PDF	

The Planck constant h is $6.626\times 10^{-34}{\;m}^{2}\mathrm{kg}/s$, permittivity of free space $\varepsilon_{0}$ is $8.854\times 10^{-12}\;C/\mathrm{Vm}$, mass of electron $m_{e}$ is $9.11\times 10^{-31}\;\mathrm{kg}$, and electronic charge q is $1.602\times 10^{-19}\;C$. The values for all the parameters used are included in the result and discussion section.
